# Association between Brachial-Ankle Pulse Wave Velocity as a Marker of Arterial Stiffness and Body Mass Index in a Chinese Population

**DOI:** 10.3390/jcdd9030075

**Published:** 2022-03-03

**Authors:** Junli Zuo, Biwen Tang, Michael F. O’Rourke, Alberto P. Avolio, Audrey Adji

**Affiliations:** 1 Department of Geriatrics, Ruijin Hospital, Shanghai Jiao Tong University School of Medicine, Shanghai 200025, China; zuo-junli@163.com (J.Z.); tbw12673@rjh.com.cn (B.T.); 2 Faculty of Medicine, Health and Human Sciences, Macquarie (University) Medical School, Sydney 2109, Australia; alberto.avolio@mq.edu.au; 3 St Vincent’s Clinical Campus, University of New South Wales Medicine and Health, Sydney 2109, Australia; m.orourke@unsw.edu.au; 4 Victor Chang Cardiac Research Institute, Sydney 2010, Australia; 5 St Vincent’s Hospital Applied Medical Research, Sydney 2010, Australia

**Keywords:** arterial stiffness, cardiovascular disease, body mass index, blood pressure, age, sex

## Abstract

Objectives: Arterial stiffness is widely accepted as an important predictor of cardiovascular disease (CVD) development. While obesity is generally associated with increased CVD risk, there is evidence that overweight patients with existing CVD may have better clinical outcomes than their lean counterparts. Our study sought to observe any potential association between brachial–ankle pulse wave velocity (BAPWV), a marker of arterial stiffness related to CVD risk, and Body Mass Index (BMI), a crude and widely used measure of obesity. Methods: Adult individuals (*n* = 857) assessed for routine CV risk were included and grouped according to their BMI (<25 kg/m^2^: normal; 25–30 kg/m^2^: overweight, ≥30 kg/m^2^: obese). Their anthropometric parameters, brachial cuff pressures, and BAPWV were measured. Results: Brachial pressure was significantly higher as BMI increased. BAPWV showed a positive linear association with systolic (r = 0.66, *p* < 0.01), mean (r = 0.60, *p* < 0.01), diastolic (r = 0.51, *p* < 0.01), and pulse (r = 0.53, *p* < 0.01) pressures. However, a linear relationship between BMI and BAPWV was only apparent in males aged <50 years (*p* = 0.01) and in females aged ≥50 years (*p* < 0.01). In individuals with similar brachial systolic pressure, BAPWV was higher in normal-weight subjects compared to overweight–obese ones. Conclusions: This conflicting finding is attributed to an overestimation of the degree of arterial stiffness as a measure of CVD risk in individuals with a less ‘healthy’ BMI. This suggests that BMI may not the appropriate obesity indicator to assess CV risk. Our finding emphasizes the importance of establishing a non-linear relationship between CVD risk, age, and BMI, taking into account apparent sex differences, to predict future CV events. While this finding may suggest a lower degree of stiffness in large arteries of overweight–obese subjects compared to their normal-weight counterparts, the potential implications for individuals with higher BMI need be explored further.

## 1. Introduction

Arterial stiffness is widely accepted as an important predictor of cardiovascular disease (CVD) development [1,2] and is usually considered as an index of vascular aging quantifiable by the measurement of pulse wave velocity (PWV). There is an abundance of information that shows a strong association between arterial stiffness and future cardiovascular events in various clinical cohorts as well as in the general population, independently of traditional risk factors [3,4]; the relative ease and feasibility of PWV measurements has made this index widely utilized in identifying CVD risk associated with arterial stiffness.

One of the methods to measure PWV is brachial-ankle PWV (BAPWV). BAPWV has been shown to be closely correlated with the directly measured aortic and carotid–femoral PWV [5]. This measurement can be performed easily using an automatic cuff for each of the four limbs by an oscillometric method [6]. Another method of assessing vascular stiffness is the ankle–brachial index (ABI), that is the ratio of the ankle systolic blood pressure (BP) to the brachial systolic BP. ABI has been reported to have an inverse relationship with the presence of CV risk factors [7]. While both indexes are shown to be markers of arterial stiffness, there are conflicting reports on how well each one predicts CV risk in the presence of different diseases [8,9]. One further limitation of PWV measurement is its dependence on blood pressure (BP) level at the time PWV is taken, thus making this parameter unsuitable to assess BP control with hypertensive therapies [10]. An alternative to PWV is the cardio–ankle vascular index (CAVI) [11]; this index is not BP-dependent and may reflect the stiffness of a section of an artery.

Obesity is a common and costly epidemiologic challenge worldwide. In China, obesity has become a major public health issue, and its prevalence has increased rapidly in the past four decades. In 2019, it was estimated that 34.3% of adults were overweight, and 16.4% of adults were obese [12,13]. Obesity is generally associated with metabolic syndrome and will lead to adverse health outcomes [14,15]. It is also associated with an increased risk of developing cardiovascular disease (CVD), especially heart failure (HF) and coronary heart disease (CHD) [16]. However, studies in overweight and obese subjects with established diseases often found their heavier weight has a potentially protective effect when it coexists with CVD; this phenomenon is termed the “obesity paradox” [17,18]. While the obesity paradox has been observed in patients with HF and CHD, recent studies reported the same observation in patients with hypertension [19], atrial fibrillation [20], and pulmonary arterial hypertension [21].

The ‘obesity paradox’ may be related to the use of the Body Mass Index (BMI) to measure obesity. The Body Mass Index (BMI), used worldwide, is a crude measure of obesity that classifies individuals based on their weight and height, identifying normal, overweight, and obese categories. There are studies that show that BMI indicates the lean body mass and may inadequately represent body fat distribution [22]—the latter is a stronger predictor of CVD morbidity and mortality [23]. Further, the practice of classification based on BMI may not distinguish body shape or body fat distribution [24]. While obesity has been known to affect both sexes, it is apparent that females who are overweight or obese have a higher susceptibility to weight-related stiffening of the arteries associated with hypertension [25], hence increased CVD risk [26].

In this study, we attempted to observe any potential association between BMI, a simple measure of obesity, and arterial stiffness measured as BAPWV, ABI, and/or CAVI, in a health assessment clinic setting.

## 2. Methods

Individuals attending an outpatient clinic for CV risk screening between April 2017 and June 2018 at Ruijin Hospital North, Shanghai, China, were invited to participate. Subjects with any CV symptoms and those with clinical or laboratory evidence of acute CV-related and/or cerebrovascular diseases in the 3 months prior to the assessment were excluded, as well as those with any malignancy. All subjects provided written informed consent. This study was approved by the Ethics Committee of Ruijin Hospital North.

Following demographic and anthropometric data collection, a total of 857 recruited subjects were divided into 3 groups based on their BMI (<25 kg/m^2^: *normal*; 25–30 kg/m^2^: *overweight*, ≥30 kg/m^2^: *obese*). Measurements of brachial cuff pressures, BAPWV, and ABI were performed in the supine position using the Omron device (BP-203RPEIII VP-1000 Kyoto, Japan), following 10 min of rest in a quiet, temperature-controlled (22 °C) room. Appropriate standard-size four pressure cuffs were wrapped on both sides of the arms and ankles to measure systolic (SP), diastolic (DP), mean (MP), and pulse (PP) pressures and simultaneously record the arterial pulse waveform to estimate BAPWV and ABI [27]. Due to the health screening protocols performed at a busy outpatient department, only single-point measurements of brachial pressure, BAPWV, and ABI were performed, and measurements were made with the participant in a stable state. During the assessment, if an individual was found to have an ABI < 0.9 and >1.3, we would perform a vascular Doppler test of lower limb arteries to confirm whether there was any asymptomatic peripheral arterial disease (arterial obstruction). We also estimated CAVI using the same BAPWV value as a substitute of PWV determination from heart to ankle [28].

Besides analyzing the whole cohort, we hypothesized that some features related to different class of BMI might be partly associated with sex, as we previously reported [25]; thus, we also grouped and further analyzed males and females separately.

All statistical analyses were performed using SPSS 24.0 for Windows (SPSS Inc., Chicago, IL, USA). A 2-sided *p*-value of <0.05 was considered statistically significant. Continuous variables are expressed as mean ± SD. One-way ANOVA with Bonferroni post-hoc test or Student’s t-test was used to detect significant differences between BMI classes. Pearson’s correlation and partial correlation analyses were used to assess the relationship between BMI and BAPWV, ABI and CAVI, adjusted for age, sex, heart rate, and MP. The association of BMI with BAPWV as a marker of arterial stiffness was assessed by univariate and linear regression adjusted for known cardiovascular risk factors (age, sex, heart rate, MP, or SP). We further applied a mediation analysis to examine the influence of age on the relationship between BMI and BAPWV using the PROCESS macro for SPSS.

## 3. Results

A total of 857 subjects (mean age 47 ± 15 years; 17–89 years; 72.6% males) were recruited; 59% of them were classified in the normal weight group (35% overweight, 6% obese) (Table 1). The obese group was the youngest (average 43 years), while the overweight group was the oldest (average 51 years). The average values of brachial pressures, BAPWV, and ABI were used for the analysis to minimize any potential difference between left and right measurement sites. Compared to individuals with normal weight, SP and DP were significantly higher in the overweight/obese group (both *p* < 0.01) (Table 1). BAPWV showed a strong linear correlation with age (r = 0.658, *p* < 0.001), SP (r = 0.621, *p* < 0.001), MP (r = 0.603, *p* < 0.001) and a positive correlation with other pressure indices (Table 2); a weaker but significant correlation with heart rate and BMI was also observed. In contrast, ABI only showed a positive and significant correlation with age, heart rate, SP, and PP (Table 2), while the derived CAVI only showed a positive and significant correlation with age, heart rate, and all pressures but not with BMI (Table 2). To understand to what the extent of the relationship between BMI and BAPWV is influenced by age, we performed a mediation analysis. We found that age (36.2%) partly mediated this relationship between BMI and BAPWV, as expected.

**Figure 1:**
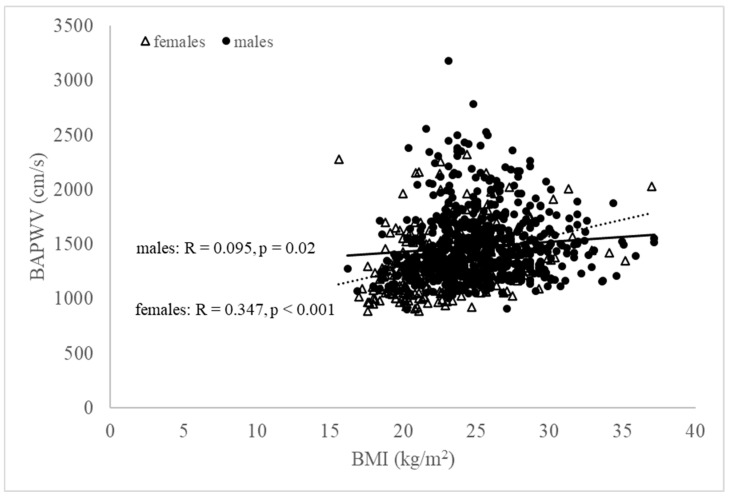
Relationship between BAPWV and BMI (black open triangles, females; black closed circles, males). The correlation between BAPWV and BMI was significantly stronger in females compared to males.

**Figure 2:**
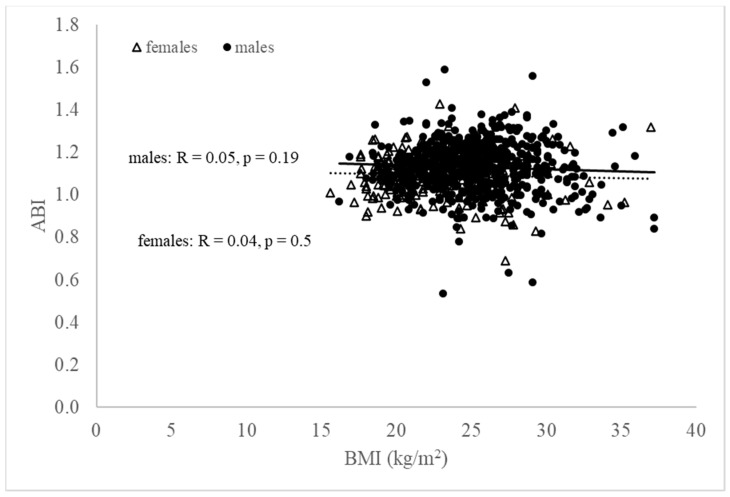
Relationship between ABI and BMI (black open triangles, females; black closed circles, males). There was a small correlation between ABI and BMI for both males and females.

Following our initial analysis of the whole cohort, we further analyzed males and females separately (Table 3, Figure 1). In males, BAPWV was highest in the overweight group, lower in the obese group, and lowest in the normal weight group, which can be partly attributed to the age of the male subjects (the oldest was in the overweight group, the youngest was in the obese group), despite brachial pressure values being all highest in the obese group (Table 3). In females, there was a strong and steady significant increase in BAPWV as BMI increased (Table 3). In our cohort, ABI was found to be not significantly different in different BMI classes for both males and females (Figure 2). Further, our study calculated CAVI as a derivation using BAPWV rather than estimating it directly; therefore, we focused on examining the association of BAPWV with BMI and other hemodynamic parameters.

**Figure 3:**
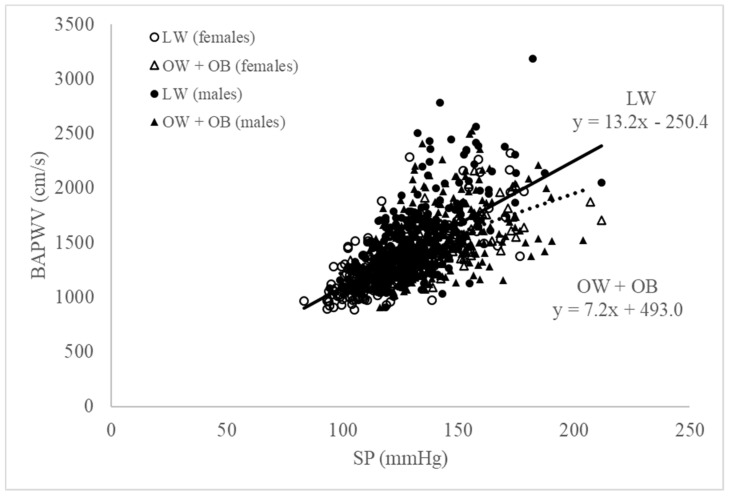
Relationship between brachial SP and BAPWV between normal-weight (LW = lean weight) (black open circles, females; black closed circles, males) and overweight (OW)/obese (OB) groups (black open triangles, females; black closed triangles, males). A linear relationship (solid line for LW, dashed line for OW + OB) between BMI class and BAPWV was only apparent for males aged ≤50 years (*p* = 0.01) and females aged >50 years (*p* < 0.01). The increase in arterial stiffness (measured as baPWV) in the normal group appeared to be greater with similar brachial SP values when these subjects were compared to individuals in the overweight and obese groups.

This apparent disparity between BAPWV and BMI with age and sex led us to further investigate the independent association of BMI with sex and with age (<50 and ≥50 years) using a simple linear regression analysis (Table 4, Figure 3). The strongest set of predictors for BAPWV were age, sex, BMI, heart rate, and SP (model 2, R^2^ 0.69), while age, sex, BMI, heart rate, and MP were the second strongest set of predictors for BAPWV (model 1, R^2^ 0.68). We found that BMI was negatively associated with BAPWV (*p* < 0.001) in all regression models. The discrepancy between males and females was evident only when age and BMI were considered as predictors of BAPWV. In younger (≤50 years) males, both age (β = 0.19, *p* < 0.0001) and BMI (β = −0.17, *p* < 0.0001) were strong predictors of BAPWV, while for older (>50 years) males, only age remained a strong predictor (β = 0.45, *p* < 0.0001). In contrast, in younger females, only age was a strong predictor of BAPWV (β = 0.28, *p* < 0.0001), while in older females, both age (β = 0.42, *p* < 0.0001) and BMI (β = −0.23, *p* = 0.004) were predictors.

## 4. Discussion

Obesity is now seen as a global health problem with increasing prevalence worldwide. A systematic analysis of the Global Burden of Disease 2013 Study [29] showed that the percentage of individuals with BMI ≥ 25 increased from 28.8% in 1980 to 36.9% in 2013 for men and from 29.8% to 38.0% for women. China’s obese population is described as ranking the second in the world after that of the United States (13%) [30]; therefore, it is appropriate to develop suitable early detection methods and interventions for obesity in China [12,13].

Our study found that all brachial pressures were significantly higher as BMI increased. BAPWV showed a positive linear association with SP (r = 0.66, *p* < 0.01), MP (r = 0.60, *p* < 0.01), DP (r = 0.51, *p* < 0.01), and PP (r = 0.53, *p* < 0.01). However, a linear relationship between BMI and BAPWV was only apparent for males aged <50 years (*p* = 0.01) and females aged ≥50 years (*p* < 0.01). When considering individuals with similar brachial systolic pressures, BAPWV was higher in the normal-weight group compared to the overweight–obese group. We attribute this disparity to an overestimation of the degree of arterial stiffness as a measure of CVD risk in subjects with a less ‘healthy’ BMI, which suggests that BMI may not be the appropriate obesity indicator to assess CV risk.

Observation at our Sydney clinic suggests that obesity in humans may “mask” arterial degeneration that is usually described in individuals with hypertension and occurs with aging. Therefore, we performed the current study to explore if any association exists between age-related and/or sex-related stiffening of the arteries measured as BAPWV with BMI in a general outpatient setting.

While obesity is a well-known CV risk factor, when obesity and HF or CHD coexist, the prognosis in patients with high BMI seems to be more favorable as compared to those who have a normal weight [18,31,32,33,34]; this fact is termed the “obesity paradox”. This finding has been attributed to an overestimation of the degree of CVD risk associated with arterial stiffness in individuals with a less ‘healthy’ BMI [23]. In fact, the MESA study investigators [35] found that a higher BMI was significantly associated with a lower degree of arterial stiffness, independently of common CVD risk factors.

The relevance of BMI to BAPWV as a measure of arterial stiffness remains controversial [36,37]. In our study, BAPWV tended to rise significantly with an increase in BMI; however, this was not clear. A recent study [38] did not find any significant correlation between BAPWV and BMI, suggesting that arterial stiffness may have a weaker association with the overall obesity status. While our study showed a linear relationship between BMI class and BAPWV, this was only apparent for younger males aged <50 years (*p* = 0.01) and older females aged ≥ 50years (*p* < 0.01). Females may be particularly affected by obesity-related CVD [26], as the natural protection by females hormones diminishes with age. Our study also found that BAPWV increased as BMI increased towards the overweight level, yet in the obese population, BAPWV decreased, although it remained higher compared to that in normal-weight individuals (Table 3). Our findings were further supported when we investigated the relationship between brachial SP and BAPWV. In overweight and obese subjects, BAPWV showed a lower-than-expected value with higher brachial SP, than in individuals with normal weight, suggesting that the normal-weight group had “stiffer” arteries (Figure 1). These conflicting results may be attributable to differences in age, comorbidities, other CVD risk factors, or even in the measurement of anthropometric and/or hemodynamic properties. Previous studies reported either a positive or a negative association between BMI and arterial stiffness and attributed this inconsistency to the arterial segment where PWV was measured, particularly in children and youth [39,40,41,42]. The weight gain reflected as an increased BMI may have a different impact on different arterial segments, and this may be dependent on BP or on passive arterial changes. Subsequently, these changes may impact on the measurement of ‘regional’ arterial stiffness markers in different stages of life, i.e., adolescence, adulthood, or mature age. Indeed, another study including middle-aged patients, with normal or high blood pressure and hypertension reported significant correlations between BMI and PWV, brachial augmentation index, aortic augmentation index, and arterial age [43]. To undertake a comprehensive analysis including these variables is beyond the scope of our observational study, whose purpose was to identify individuals in a general outpatient clinic with a greater risk of CVD due to their weight and BMI.

PWV has been shown to be an indicator of arterial stiffness and CV risk; a 1 m/s increase in PWV is associated with a 14% increased risk of CV disease [44]. PWV measurement is currently the optimal measurement of arterial stiffness because of its accuracy, simplicity, predictive value, and reproducibility. The Malmö Diet and Cancer Study attempted to find the association between PWV and inflammation for CVD risk prediction among elderly participants [45] and showed that the measurement of PWV can be used as an objective and robust indicator of CVD risk. They suggested that low-grade systemic inflammation might be the missing link between different metabolic phenotypes and CV risk [46]; this warrants further consideration. The MESA study [35] found that the inverse relationship between arterial stiffness and BMI remained in the elderly cohort, suggesting that arteries in elderly individuals with normal weight may be less stiff than they appear by measuring BAPWV. Another study found that regardless of the BMI, females are generally more affected by progression of arterial stiffness [47], while this is not apparent in males. The latter may lend support to other studies which have found BMI to be a crude measures of total adiposity which may inadequately represent body fat distribution and/or body shape [22]. A potential explanation of the obesity paradox highlights the inherent limitations of assessing adiposity by BMI alone. As most studies usually categorize patients based on their BMI, the groups that showed benefit may have a relatively healthier fat distribution and a higher muscle mass [48]. It is also hypothesized that unaccounted for confounding factors like cardiorespiratory fitness may be responsible for the obesity paradox [49].

A major limitation of our study is that females were under-represented, only comprised about 27.5% of the total study population, and only 7% of them were in overweight or obese. However, the results are consistent with our findings previously published [25], showing that overweight and obese females are more susceptible to weight-related hypertension associated with faster degeneration of the elastic arteries. Furthermore, the current study was an observational study and did not examine the effect of a greater BMI on endpoint events. We did not analyze other cardiovascular and metabolic risk factors or any cardiovascular-related medication. We also did not analyze body composition, which would be useful to discriminate to what extent ‘fat excess´ contribution to a person’s BMI is associated with arterial stiffness, as well as any inflammatory biomarkers which may be predictors of PWV. Lastly, the study population was mainly recruited from a health assessment clinic in Shanghai, China; hence, the findings may not be applicable to other ethnicities.

The prognostic value of arterial stiffness in the future development of CVD has been established. With the increasing prevalence of obesity worldwide, establishing the associating between arterial stiffness and obesity will have significant clinical importance for the early detection and prevention of CVD. This is particularly valuable for obesity treatment, which must consider the strong link between indicators of obesity and CV risk. It is apparent from our study and others that BMI, an overall obesity indicator, may not reflect adipose tissue distribution, which has a strong correlation with CVD risk. A thorough control of CV risk factors such as arterial stiffness and hypertension, metabolic syndrome, and dyslipidemia contributing to obesity is vital to reduce future CVD events. Our preliminary findings emphasize the importance of establishing a non-linear relationship between BMI, age, and CV risk, taking into account apparent sex differences. Further investigation on the effect of age and sex, considering the degree of obesity and other anthropometric indices, as well as their clinical implications, is warranted.

## Figures and Tables

**Table 1:** Subjects’ characteristics grouped according to their BMI (mean ± SD).

	LW BMI < 25 kg/m^2^	OW BMI 25–30 kg/m^2^	OB BMI > 30 kg/m^2^	*p*-Value (OW vs. LW)	*p*-Value (OB vs. LW)
N	503	300	54		
Age (years)	45.9 (15.3)	50.5 (14.4)	42.7 (14.7)	<0.001	NS
SP (mmHg)	125.1 (18.0)	137.7 (17.2)	155.9 (17.6)	<0.001	<0.001
MP (mmHg)	94.1 (14.7)	103.9 (14.2)	117.2 (15.6)	<0.001	<0.001
DP (mmHg)	75.7 (10.9)	82.9 (10.8)	92.2 (13.5)	<0.001	<0.001
PP (mmHg)	49.4 (10.7)	54.7 (10.8)	63.7 (10.1)	<0.001	<0.001
BAPWV (cm/s)	1403 (333)	1505 (295)	1499 (229)	<0.001	NS
ABI	1.12 (0.1)	1.13 (0.13)	1.09 (0.12)	NS	NS
CAVI	4.3 ± 1.8	4.5 ± 1.8	4.0 ± 1.8	0.033	0.090

BMI = Body Mass Index, LW = lean (normal) weight, OW = overweight, OB = obese. SP = Brachial Systolic Pressure, MP = Brachial Mean Pressure (by integration), DP = Brachial Diastolic Pressure, PP = Brachial Pulse Pressure, BAPWV = brachial–ankle Pulse Wave Velocity, ABI = ankle–brachial index, CAVI = cardio–ankle vascular index, NS = not significant.

**Table 2:** Correlation between BAPWA (top) and ABI (bottom) with age, BMI, and hemodynamic parameters.

	**Age**	**BMI**	**HR**	**SP**	**DP**	**MP**	**PP**
**BAPWV**							
CC	0.658	0.206	0.198	0.621	0.513	0.603	0.532
*p*-value	<0.001	<0.001	<0.001	<0.001	<0.001	<0.001	<0.001
	**Age**	**BMI**	**HR**	**SP**	**DP**	**MP**	**PP**
**ABI**							
CC	0.293	−0.005	−0.165	−0.165	−0.003	−0.032	−0.158
*p*-value	<0.001	NS	<0.001	0.008	NS	NS	<0.001
	**Age**	**BMI**	**HR**	**SP**	**DP**	**MP**	**PP**
**CAVI**							
CC	0.666	0.046	0.175	0.31	0.196	0.3	0.325
*p*-value	<0.001	NS	<0.001	<0.001	<0.001	<0.001	<0.001

CC = Pearson’s correlation coefficient, *p*-value NS = not significant. BAPWV = brachial–ankle pulse wave velocity, ABI = ankle–brachial index, CAVI = cardio–ankle vascular index. BMI = body mass index, HR = heart rate, SP = systolic pressure, DP = diastolic pressure, MP = mean pressure, PP = pulse pressure.

**Table 3:** Subjects’ characteristics according to their BMI for males (top) and females (bottom) separately.

	LW BMI < 25 kg/m^2^	OW BMI 25–30 kg/m^2^	OB BMI ≥ 30 kg/m^2^	*p*-Value (LW vs. OW + OB)
**Males**				
N	323	254	45	
BMI	22.7 (1.7)	27.0 (1.4)	32.1 (1.9)	<0.001
Age (years)	47.9 (15.8)	50.6 (14.5)	41.2 (14.4)	<0.001
SP (mmHg)	127.6 (15.9)	136.1 (15.2)	156.2 (18.7)	<0.001
MP (mmHg)	95.8 (13.1)	102.7 (12.8)	117.7 (16.5)	<0.001
DP (mmHg)	77.4 (9.8)	82.7 (10.4)	92.8 (13.8)	<0.001
PP (mmHg)	50.2 (10.2)	53.4 (9.7)	63.4 (10.2)	<0.001
BAPWV (cm/s)	1454 (338)	1509 (299)	1477 (212)	0.107
CAVI	4.5 (1.9)	4.6 (1.8)	3.8 (1.1)	0.016
**Females**				
N	173	54	9	
BMI	21.1 (1.9)	26.6 (1.2)	32.5 (2.4)	<0.001
Age (years)	41.8 (13.8)	49.6 (14.1)	49.7 (15.0)	<0.001
SP (mmHg)	120.1 (20.5)	144.4 (23.1)	154.6 (11.1)	<0.001
MP (mmHg)	90.6 (17.0)	108.3 (18.4)	115.4 (10.5)	<0.001
DP (mmHg)	72.2 (12.0)	83.5 (13.0)	89.0 (12.3)	<0.001
PP (mmHg)	48.0 (11.6)	60.9 (13.1)	65.5 (9.6)	<0.001
BAPWV (cm/s)	1302 (300)	1481 (276)	1610 (291)	0.007
CAVI	3.9 (1.5)	4.1 (1.2)	4.6 (1.8)	0.176

BMI = Body Mass Index, LW = lean weight, OW = overweight, OB = obese, *p*-value was the significance level between LW and OW + OB due to the low numbers. SP = Brachial Systolic Pressure, MP = Brachial Mean Pressure (by integration), DP = Brachial Diastolic Pressure, PP = Brachial Pulse Pressure, BAPWV = brachial–ankle Pulse Wave Velocity.

**Table 4:** Determinants of BAPWV explored using a simple linear regression analysis.

Variable	β	*p* Value	R²
**Model 1**			0.679
Age (years)	0.556	<0.001	
Sex (female vs. male)	−0.077	<0.001	
BMI (kg/m²)	−0.104	<0.001	
HR (bpm)	0.183	<0.001	
MP (mmHg)	0.473	<0.001	
**Model 2**			0.694
Age (years)	0.540	<0.001	
Sex (female vs. male)	−0.075	<0.001	
BMI (kg/m²)	−0.137	<0.001	
HR (bpm)	0.199	<0.001	
SP (mmHg)	0.507	<0.001	
**Model 3**			0.588
Age (≥50 vs. <50 years)	0.461	<0.001	
Sex (female vs. male)	−0.097	<0.001	
BMI (≥25 vs. <25 kg/m^2^)	−0.069	<0.001	
HR (bpm)	0.175	<0.001	
MP (mmHg)	0.49	<0.001	
**Model 4**			0.605
Age (≥50 vs. <50 years)	0.449	<0.001	
Sex (female vs. male)	−0.192	<0.001	
BMI (≥25 vs. <25 kg/m^2^)	−0.088	<0.001	
HR (bpm)	0.192	<0.001	
SP (mmHg)	0.516	<0.001	

BMI: body mass index; SP: systolic pressure; MP: mean pressure; BAPWV: brachial–ankle pulse wave velocity; HR: heart rate.

## Data Availability

The data underlying this article cannot be shared publicly due to the Privacy of individuals that participated in the study.
